# Abstract of the Proceedings of the Buffalo Medical Association

**Published:** 1858-04

**Authors:** Austin Flint


ART. II.—*Abstract of the Proceedings of the Buffalo Medical Association*

TUESDAY EVENING, Feb. 16, 1857.

The regular meeting of the society on the first Tuesday in February was adjourned to Wednesday evening.

February 10th. Owing to the death of one of its most respected members, Dr. Lewis, the meeting was adjourned to date.

The association met.

Present—The President, Prof. Flint, in the chair; and Drs. Newman Gay, Wilcox, White, Miner, Lemon, Rochester, Whitney, Nichols, Flint, Jr., Treat, Hamilton, Wyckoff and Rankin.

A reading of the minutes of the last meeting was called for, but as they were not present, it was dispensed with.

The committee on Puerperal Convulsions was prepared to report, but the reading was deferred until later in the evening.

*Dr. Rochester,* committee on Tracheotomy in Membranous Croup, asked further time, which, on motion, was granted.

*Prof. White* exhibited specimens of growths which he had removed from the interior of the uterus. The patient was a married female, aged about 40 years, who had never borne children. She had been affected with a continual and exhausting hemorrhage from the uterus for a long time. On a careful examination with the speculum, there was discovered a small uterine polypus about the size of a bean: this was removed, and the patient assured of recovery; the after treatment consisting of astringent injections with the internal use of ferruginous tonics. Under this treatment the patient improved, but a discharge continued, being about one-half as copious as before the removal of the polypus. A specular examination discovered nothing at the mouth of the uterus; but its cavity was found to be enlarged and the lining membrane roughened, by an exploration with the uterine sound. Iodine was passed up into the cavity, but without any effect on the discharge; and careful examination revealed vegetations or polypoid growths from the mucous membrane. Dr. White determined to remove these with Recamier's instrument, which he had before used with success for a similar purpose; (this instrument is merely a scoop, of a size sufficiently small to admit of its introduction into the uterus,) he accordingly passed it into the cavity of the organ, and thoroughly scraped its internal surface, removing a number of excrescences, varying in size from that of an apple seed to a millet seed. These came away easily, and it is probable that they were all removed. This succeeded in entirely arresting the hemorrhage.

A specimen of these excresences was examined microscopically by Dr. Flint, Jr., who found them to consist of the uterine mucous membrane with some areolar tissue.

Dr. White added, that in his opinion, these excrescences often existed in cases of uterine hemorrhage, and that the best means of arresting it, was the removal of the growths by means of Recamier's instrument.

Dr. Rochester enquired if any topical application was to be made after the operation.

Dr. White replied that he applied the muriated tincture of iron. The operation is not attended with much hemorrhage, and there did not seem much liability to return. The discharge was not continually sanguinolent, but when it did not consist of blood, it was watery and not purulent. Dr. White has no doubt but these growths are analagous in their character to ordinary uterine polypi. In making topical applications to the interior of the uterus, Dr. W. uses a bit of lint, attached to a piece of whalebone, or grasped by a pair of long forceps, saturated with the requisite fluid. He prefers this method to injection into the body, as he has seen cases where these injections were followed by most intense abdominal pain, even so severe as to cause a suspicion of peritonitis; the fluid might indeed have passed through the fallopian tubes into the peritoneal sac. He had repeatedly arrested obstinate uterine hemorrhage by passing up a dossil of lint saturated with the muriated tincture of iron.

*Dr. Treat* mentioned the practice followed by Prof. Miller in such cases, which consists in passing up a strip of linen saturated with some astringent fluid, with a probe, and allowing it to remain.

Dr. Rochester remarked that he had always been unable to pass the strip sufficiently far into the cavity of the uterus, while he could always make applications in the method mentioned by Dr. White, with great facility.

Dr. White thought, that in passing up a strip of cloth, in the manner recommended by Dr. Miller, most of the fluid would be squeezed out in passing it through the os uteri.

*Dr. Rochester* presented an account of a case of supposed Pyæmia.

### *Report of a Case of Supposed Pyœmia.*

On Monday, Feb. 1st, Dr. M. G. L., of Black Rock, was exposed to a cold and piercing wind for a long time. On reaching home he was chilled through. The following day, Feb. 2d, he was confined to the house, but not to his room.

Wednesday, Feb. 4. He was still more ill, and had two distinct rigors, followed by imperfect reäction; he also experienced sharp pain in the left scapular region and left arm, which was aggravated by motion. He took eight grains of calomel with ten of rhubarb, which operated freely.

Thursday, Feb. 4th. At his request I visited him about noon. He was lying upon his right side, and could neither move nor be moved without suffering excruciating pain. With much care and difficulty his clothing was sufficiently removed to afford a view of the back. A distinct swelling, extending from the left upper scapular to the left lumbar region, was apparent. There was no redness of the skin. Palpation of the swollen parts was impracticable, on acconnt of the excessive agony induced by the slightest manipulation. When perfectly quiet there was no pain, nor was it produced by a full inspiration. It appeared to be confined to the integument. Physical exploration, carefully conducted, afforded no indications of intrathoracic disease.

*General Symptoms.* Surface sallow, especially that of the face, dry and evincing slight febrile movement; lips and nails somewhat livid; gums pale, spongy, and bleeding on slight pressure; (the Doctor attributed the state of the gums to the mercurial he had taken the night before, as he was unusually susceptible to its action,) tongue moist, edges and tip pale, centre and posterior thickly furred. Tonsils and fauces pale, flabby, and covered with a white, tenacious, frothy mucus. From one nostril there was a slight discharge of a muco-sangninolent fluid: this had been observed at intervals for several weeks.

Pulse 120, and very feeble. Thirst present, but not urgent; no appetite; urine normal.

*History.* Health good until August last, when he had an attack of dysentery, which he attributed to an impure cistern near his sleeping apartment. This was succeeded by a low form of intermittent fever, which was again followed by dysentery. He was visited in this illness by Prof. Flint. Dating from this period, he has never been entirely well, being frequently annoyed with flatulence, colicy pains, and diarrhœa. He has likewise had tonsilitis, sore and swollen lips, offensive breath, and enlargement of the cervical glands. Early in October he suffered from severe and obstinate furuncular inflammation of the nose and upper lip, attended with spontaneous salivation, which continued for nearly a month. Two weeks ago he lost his only child with measles: it was ill for ten days, and during its sickness, the father was its constant and untiring nurse.

The death of the infant was a severe blow, but notwithstanding the consequent mental depression, and the physical weakness induced by anxiety and sleepless nights, he returned to his professional avocations, in which he was busily engaged when attacked by his last illness.

*Diagnosis.* The pain and swelling in the back directed especial attention to that locality, and in my first interview, fear was expressed that it indicated deep seated muscular abscess, but on account of the extreme sensibility of the skin, it was hoped that an herpetic, or erythematous eruption, would soon appear, and relieve and explain the excessive local tenderness. The constitutional symptoms could readily be attributed to the sharpness of the attack, considering the preceding ill health, and recent affliction of the Doctor.

If there was doubt, however, respecting the diagnosis, there was none as to the requisite treatment. Warm laudanum, covered with a hot fomentation, was directed as a local application, and the following prescription was made:

℞. Quiniæ sulph. grs. ij.
Pul. Doveri, grs. vj.
Camphoræ gr. ss.

To be repeated every three hours. Also to take beef essence and other nourishment freely.

Feb. 5th, 11½ A. M. Local tenderness not abated. General condition improved. Pulse 100. Surface moist; has had a quiet night, and perspired freely. Arm less painful, and not at all swollen, but the pressure even of a feather upon the back could not be tolerated. Abdomen tympanitic, much flatulence, but no griping, and no stool. Urinary secretion abundant. Diected general treatment continued, and, as a local anæsthetic, the vapor of carbonic acid.

Feb. 6th, 9 A. M. General aspect improved; local tenderness and swelling diminished, but not sufficiently to allow manipulation. Pulse, however, more frequent, (116) and feeble. Ringing in the ears, and slight deafness. This was attributed to the quinine, of which he had now taken about forty grains.

Directed: Tr. iodini comp. locally, and the quinine; Dover's and camphor powders to be taken every six hours; beef essence and strong milk punch, *ad libitum*. Also, at the Doctor's request, one seidlitz powder.

I was unable to make another visit in the afternoon, being occupied with a severe case of uterine hæmorrhage.

Feb. 7th, 10½ A. M. Has passed a bad night. The bowels have moved copiously five or six time, as the Doctor supposed from the seidlitz powder, of which he took but half. Pulse 120, and very feeble; surface moist and cadaveric; lips swollen and livid. Local tenderness and swelling much less. Distinct fluctuation on palpation. The Doctor had taken twelve grains of Dover's powder, with the effect, as he thought, of controlling the hypercatharsis.

Directed a warm poultice to the swollen part, and to take beef essence and brandy very freely, and in case of a return of the diarrhœa, ℥ s. of paregoric.

10 P. M. Prof. White in consultation. Patient much worse, has had very copious colliquative diarrhœa throughout the day; has refused stimulants and nourishment. Pulse very feeble and intermittent; skin cool and clammy. Mind wandering, with lucid intervals. Local tenderness very much lessened.

Prof. White concurred with me as to fluctuation on pressure, and also as to the probability of purulent absorption, from the gravity of the general symptoms.

Stimulants and nourishment were directed to be pushed. Brandy ℥ s. and beef essence ℥ s. every half hour, if the stomach would tolerate them. Also enemata of starch with ʒj. of laudanum, and ten grains of tannin after each evacuation. Also morphine in half grain doses by the mouth, if the injections should not restrain the stools. These directions were faithfully carried out by Dr. Sylvester Rankin, who remained with Dr. L. during the remainder of his life.

Feb. 8th, 4½ A. M. Prof. Flint in consultation. Patient rapidly failing. Stools very frequent and involuntary.

Stimulants and morphine continued.

9 A. M. Sinking.

4½ P. M. Died.

*Autopsy*, 23 hours post mortem.

Present—Drs. Flint, Wilcox, Newman, Wyckoff and Rankin.

It should be noted that this post mortem examination was made at the request of the patient.

Cadaveric rigidity slight; skin sallow. Posterior portion of the body very dark from sanguinolent imbibition: this was observed several hours before death.

*Sectio Cadaveris.* Conducted by Dr. A. W. Nichols. Two deep incisions, crossing each other at right angles, were made in the left scapular region, *No pus* was detected, but the whole muscular tissue was softened and infiltrated with a sero-sanguinolent gelatinous fluid.

*Head.* Not examined.

*Thorax.* Left lung, one or two chalky concretions in the apex, marked *hypostatic congestion* of posterior portion; otherwise healthy. Right lung, adhesions from old pleurisy; in all other respects corresponding with the left lung.

*Pericardium.* Contained about an ounce of serum.

*Heart.* Amount of fatty deposit rather large, especially at apex; walls of left ventricle three quarters of an inch thich; central cavity small. (Prof. Flint thought this might possibly be due to concentric hypertrophy.) Valves normal.

*Liver.* Slightly enlarged; healthy.

*Spleen.* About three times the usual size, and a little softer than common.

*Kidneys.* No morbid changes.

Neither the stomach, bowels or mesenteric glands presented any evidences of disease.

*Blood.* Unusually fluid.

The lateness of the hour, and other circumstances, precluded an examination of the joint cavities, and other portions of the body.

*Remarks.* Pathologists recognise two distinct forms of pyæmia. The one *primitive*, originating in the blood itself, the other *secondary*, resulting from absorption of purulent matter. The first form is the more surely and rapidly fatal, producing necræmia, and consequent destruction of life, the morbid changes being mostly confined to the circulating fluid. Purulent depots are not always found in either variety, and rarely, in fact, in primitive pyæmia. When not detected elsewhere, they are often discovered in the joint cavities, especially in those of the knee and shoulder.

In the case reported, neither the contents of the cranium, nor of the joint cavities were examined; it cannot, therefore, be said that there were no pus depôts: but even had these *not been* detected after a *complete and critical* examination, the absence of disease in the vital organs, sufficient to account for the symptoms presented during life, would strengthen the idea, and as far as negative evidence can go, establish the diagnosis of pyæmia. The condition, as found in the muscular structures of the left scapular region, would doubtless have eventuated in abscess, had the patient possessed more vigor, and by this channel the blood poison might have been eliminated. Taking into consideration the history of the case, the writer is inclined to trace the source of the pyæmia to the dysenteric attack in August last. This it will be remembered was followed by "a low form of intermittent fever." Prof. Flint, who visited Dr. L. in this illness, informs me that "it was not a frank, well defined intermittent."

Hepatic abscess is recognised as an occasional sequence of dysentery. On the cessation of the fever, dysentery again appeared, and it may be that its second occurrence was a natural effort, partially successful, to remove pus from the liver, or other parts of the system (vid. Rokitansky infra.) Succeeding the dysentery, we have constant ill health, colicy pain, flatulence, diarrhœa, sallow countenance, tonsilitis, enlargement of cervical glands, furuncular inflammation of lip and nose, ichorous discharge from nostril, spontaneous salivation, and other evidences of a bioken down constitution; lastly great mental and physical depression, and a fatal crisis at length evolved, immediately induced by long exposure to inclement weather. A few quotations from Rokitansky, in relation to pyæmia, and bearing upon the case reported, will close this paper. The *italics* are introduced as substantiating some of the views expressed above:

"*Pycæmia—Pus Blood.*"

"This crasis represents a *local pus production*, and also a *spontaneous primitive pycæmia* of the entire blood mass."

"The more intense the aggravation of the crasis, the less do we encounter the aforesaid depôts." * * * "In the *highest grade* extensive passive stases affect the decomposed blood, producing necrosis, with dark and *hæmorrhagic imbibition* of the textures." * * *

"The *lungs* are the especial seat of hypostatic congestion, with a dark coloration, verging upon cherry red or upon brown." * * * "It is certain that a large proportion of bland pus, taken up into the circulation, proves far less mischievous than an incomparably smaller quantity of *purulent ichor.*" * * "Pyæmia generally proves fatal. * * Inconsiderable grades of it, however, are susceptible of cure. This occurs, without doubt, partly through a conversion of the pus analagous to the metamorphosis of fibrin—partly through the elimination of the pus in *exudatory processes*, especially upon extensive mucous membranes like that of the *intestinal tract.*"

To the above may be added, from Valleix, in the article entitled "*Infection Purulente*":

"In very grave cases there has been a *yellow tint of the skin*, which has some analogy with icterus, and to which M. Maréchal attached great importance."

In reply to Prof. White's inquiry, as to the analogy of the present case with that of Dr. J. Kearney Rodgers, reported by Dr. Hosack, the writer has observed many resemblances. Dr. Rodgers was indisposed for several months preceding his last illness, suffering from diarrhœa and colicy pains. At last he was taken severely ill with what he and all his attending physicians, except Dr. Hosack, supposed to be intermittent fever. He had rigors, yellow skin, feeble and frequent pulse, with surface sometimes dry, but generally moist. A post mortem examination, conducted by Dr. Isaacs, revealed lymph and pus in the abdominal cavity, the liver was unchanged in external appearance, but on section minute drops of pus exuded from the cut surfaces. These were found to proceed from the ramifications of the portal vein. Pus was also found in the mesenteric veins. Dr. Rodger's illness was more protracted than Dr. L.'s: he lived just one month after the severe symptoms were declared. Had his disease been of shorter duration, pus might not have been detected.

*Prof. White* remarked that he had no doubt as regards the swelling mentioned by Dr. Rochester, that it was not an abscess; he thought that it was the second form of pyæmia mentioned by Rokitansky; he did not consider that that there was sufficient vitality in the system for the formation of pus: it seemed to him that the symptoms could be accounted for in this manner.

*Dr. Gay* said that he had expressed an opinion with respect to this case, that the disease was erysipelas. Dr. G. had three cases of this disease, in which the symptoms were much the same as in the case reported by Dr. Rochester. Since the reading of a full report of the case, however, he was disposed to agree with Dr. Rochester in the diagnosis; his cases occurred during an epidemic and between them and the one reported, there were many points of similarity; if this case were one of pyæmia, it was possible that his cases were of a like character. Dr. Gay gave a brief account of some of the symptoms in the cases to which he referred.

*Prof. Rochester* thought that the cases mentioned by Dr. Gay were cases of erysipelas, but that his case was of a different character, as shown by decided changes in the blood, which were indicated by many of the symptoms.

*Dr. Gay* inquired if the absorbtion of pus would produce delirium and death so speedily.

*Prof. White* answered that it would, as was abundantly shown in some forms of puerperal fever, which were dependent on the absorbtion of matter. The idea that the disease, in the case of Dr. Lewis, was erysipelas, had never occurred to him; there was no disease of that kind in the vicinity. There is no disease, in the opinion of Dr. W., which is so rapidly fatal as pyæmia.

*Prof. Hamilton* remarked, that as a rule, the physicians in attendance were most able to judge of the nature of a disease; that his knowledge of the attainments and skill of the attending physicians in this case, was such as to inspire him with the most implicit confidence in the correctness of the diagnosis; he does not see, from the report, what other conclusion could be arrived at. Dr. Hamilton mentioned a case which had come under his own observation, in which he had removed the penis for epithelial cancer. The wound had nearly healed, when the disease was reproduced in the stump. He then made a second operation, removing the entire organ; for two or three days the wound did well, discharging healthy pus, when on the fourth day, the suppuration changed in its character, the patient became very much depressed, had severe chills, and an approach to coma; he died on the third day after this change. A post-mortem examination was denied, but Dr. H. had no doubt that death resulted from purulent absorbtion.

*Prof. Flint* remarked that the case of Dr. Lewis was undoubtedly one of pyæmia, though it might seem at first to admit of discussion. In regard to Dr. Gay's cases, it would seem more reasonable to regard them as cases of erysipelas. Still the analagous symptoms in the cases might be explained by the fact that cases of malignant erysipelas often involve pyæmia, which would give rise to the symptoms described by Dr. Gay. Dr. F. thought that it was probable that pus-blood occurred in the cases mentioned by Dr. Gay, in connection with the attack of erysipelas. The pathology would be the same as in the cases of child-bed fever mentioned by Dr. White.

While in London Prof. F. attended a post-mortem examination by Prof. Jenner, the details of which we quote from his note book: "Dr. Jenner invited me to attend a post-mortem examination at the hospital, in an interesting case which was admitted into the surgical ward for prolapsus ani. After his admission he presented febrile movement and delirium, and the case was transferred to the medical ward as a case of typhus fever. Dr. J. said he had considered the case to be one of *pyæmia.* He said it had happened several times to him to see cases of pyæmia which had been called typhus; and a gentleman connected with the surgical side of the hospital said that patients with pyæmia were not unfrequently admitted as cases of rheumatism. Dr. J. told me that the pulse in this case was very frequent. I asked him if he would not consider a case probably one of pyæmia, in which the pulse was very frequent, the patient prostrated and in an alarming state, the disease not being fever, nor the symptoms otherwise accountable? He replied in the affirmative.

"When I arrived at the post-mortem room—the same in which I had met Dr. Walshe—I found he was just finishing the examination of the heart, and this was the first organ examined. He dictated notes recorded by an assistant, in the same mode as was pursued by Dr. Walshe. He measured the width of the aorta and pulmonary arteries just above the valves, and the length of the base of the mitral and tricuspid valves, opening the ventricles freely for that purpose. The heart was normal.

"The right and left lungs were then successively removed and examined. Nothing found but congestion, some sub-serous ecchymoses and a few small calculi just beneath the pleura.

"Head next opened; nothing especially abnormal found; appearances noted in full; no special pains taken to observe arachnoid effusion. Dr. J. looked into the base of the skull after the brain was removed and detected 'about the usual quantity of arachnoid effusion.'

"Liver presented nothing abnormal, nor the spleen or kidneys. The intestines (ileum) presented visible Peyer's glands, and some submucous extravasation in spots."

Where were the evidences of the correctness of the diagnosis?

Dr. J. opened the knee-joints, and in the right joint was found a considerable quantity of purulent looking matter. The membrane was injected. He opened the ankle, wrist, elbow, shoulder and hip joints. A little purulent fluid was found in the cellular tissue about one elbow joint, and a small quantity, either within, or in the neighborhood of one of the hip joints.

This was considered sufficient to settle the diagnosis—for it is evident the patient did not die from the joint affection. The latter, then, were only results of some more general disease—the blood disease—but "suppose," said Dr. Jenner, "we had not found the purulent collection? I would have been equally satisfied that the patient died with pyæmia, but could not have satisfied others to that effect."

Dr. Flint had met with cases in females after labor, and cases disconnected with labor, which presented symptoms of pyæmia.

*Dr. Flint, Jr.*, read a report of a case of hydrophobia. The case was reported to the association by Dr. Baker, but the Dr. was unable to be present and requested Dr. F. to read the case for him.

### *Case of Hydrophobia.*

About the first of October last, Charles Hoyt, aged 13 years, was bitten on the end of his nose by a dog. There were three cuts in the skin, which bled profusely. His father dressed the wound with adhesive plaster, and in a few days it was healed. Whether the animal was rabid or not is not known. Its owner said the dog was perfectly healthy and had never shown any symptoms of disease—rabies or other. But having bitten another lad a day or two after, he was killed. No anxiety seems to have been felt as to the result, and the circumstance had been forgotten by the family.

On Saturday, the 23d inst., Charles had fallen into the Creek and completely saturated his clothes with water. In this condition he kept around the streets, for some time posting bills, and finally, went into the house of an acquaintance, dried himself and went home. His family knew nothing of this until the day of his death. His health was good up to Wednesday night, the 27th inst. That night his grandfather occupied the bed with him, but could not sleep on account of his restlessness. Thursday morning, when called to breakfast, he declined eating, and said he had no appetite. As morning advanced, complained of headache and nausea. Toward noon vomited freely several times, a greenish bitter matter. Most of this day was confined to bed. Toward evening his mother gave him a dose of laxative bitters, of domestic manufacture. During the night he was more restless and complained of his head; was quite thirsty, but did not like to drink as it "took his breath away." His difficulty of swallowing arose not from any soreness of the throat, but a fear that it would suffocate him.

About 1 o'clock Friday morning, his medicine acted on the bowels, and he appeared somewhat relieved, and slept for two or three hours. When he awoke his headache had left, but he had a high fever, which lasted through the day; would call frequently for drink, which he took from a spoon; said he could n't drink from a cup or tumbler, "it took away his breath." A part of the night of Thursday and during Friday, would rouse with a start and scream, and appear frightened as though something was after him. Friday afternoon these symptoms increased in severity, and any sudden or unexpected motion made near him, would cause him to jump or spring with a cry of alarm. About 4, P. M., of Friday, he was sitting in a chair near the stove, when an elder brother came up behind him, and lifted a corner of the blanket to cover his legs; he sprang from his seat and fell to the floor, insisting that his brother threw him out of the chair.

At 5 o'clock, P. M., of this day, the 29th, Dr. Lockwood saw him gave him a large dose of calomel, and directed a tea of wormwood for drink. The calomel acted freely as cathartic and emetic. He threw up large quantities of bilious matter. He was more quiet after the operation of the medicine. Previous to the operation of the calomel his symptoms were much the same.

Saturday morning he was thought to be much better; was quiet and cheerful; partook of a little coffee and toast water. There was less difficulty in deglutition, though he had yet to take his drinks from a spoon. Towards noon of this day, 30th, began to manifest more wildness and incoherence; would suddenly jump from side to side of his bed. He said he did this to "catch his breath" which was getting away from him. Was almost constantly spitting and wiping his tongue and lips. His family had noticed, for two or three weeks, a habit of frequent spitting.

At 3 o'clock, P. M., I first saw him. An hour before had a severe chill, but now his skin was warm and moist; countenance wild and anxious; motions quick and jerking; his arms folded tightly across his chest. Complained of his heart hurting him, by thumping so hard. Considerable distress of stomach; larynx slightly sore, but did not hurt him in swallowing. Tongue but little coated; pulse nearly normal in force and frequency. Said his head did not ache. Olfactory nerves remarkably sensitive to odors of all kinds, especially previously favorite perfumery.

Prepared a weak solution of carb. ammonia, hoping it might relieve the distress of stomach, and directed warm fomentations to chest and bowels.

I suspected hydrophobia, but preferred seeing his medical attendant before prescribing. At my request, his father offered him a teaspoonful of the solution, which he at first declined, saying: "I can't take it, it will choke me; it won't go down any further than this," placing a finger on the top of the sternum. After a little persuasion, he consented to try. Wiping his tongue violently two or three times with a cloth, he rose with a jerk to a sitting posture, saying, "let me get ready." Bracing himself, he suddenly opened his mouth very wide. As the spoon touched his tongue his lips closed on it, and instantly threw himself back on the pillow, panting as though he had been engaged in violent exercise.

Closely questioning his father regarding the boy's ntecedents, I learned what has been related of the bite. There was to me no longer a doubt as to the diagnosis. I at once reported the case to Dr. L. with a request that he would resume the care of the patient. His engagements precluding this, he desired me to invite Dr. Flint to see him with me. At 5, P. M., Dr. F. and myself visited him. The spasms and glottis were now more frequent and severe. Would answer questions promptly and correctly. His general appearance much as when I saw him before. Pulse but little accelerated. Capillaries of skin much congested. Fauces and tonsils slightly inflamed.

At Dr. F.'s request, he took a teaspoonful of water, swallowing it rapidly with a jerk. The sight of water in a tumbler, and pouring it from the spoon neither excited nor disturbed him. To the questions, does it look good; would you like more of it? he promptly responded, no Sir. He kept his arms lightly folded across the chest, and almost constantly wiping his tongue and lips with each hand alternately.

Dr. F. coinciding in the diagnosis, it was decided to administer a teaspoonful of laudanum, to procure, if possible, an anodyne effect, and be guided in in subsequent treatment by the character of symptoms.

7½, P. M., Dr. Flint, Jr. and Mr. Mason, saw the patient with me. Had retained laudanum in the stomach about half an hour, when he vomited freely. Had taken some nourishment, which was also immediately thrown up. The spasms were less severe, though very restless, tossing about constantly. Perspired freely; his shirt was thoroughly wet with it. Pulse had changed to a wiry, frequent pulsation, striking about 120. It was impossible to time it exactly.

Directed a half teaspoonful of laudanum to be repeated at once if rejected. To administer nourishment and stimulants if he would take them. Within an hour, two doses were given and both rejected. His stomach would retain nothing.

9, P. M. Mr. Mason, Drs. Cushing and Stevens, at request of Dr. Flint and myself, attempted to administer chloroform by inhalation. To this the patient obstinately and peremptorily objected. He did n't like the smell. They wanted to poison and kill him.

By main force one or two inspirations were received by him, but without perceptible effect.

10, P. M. Dr. F. and myself again visited patient. Symptoms had rapidly progressed. Presented the same wild appea ance. Thoughts more incoherent, and he was quiet hardly an instant, throwing himself from one position to another. Secretion of saliva more abundant, and was very troublesome to him. Pulse at wrist not perceptible. Skin cold, and covered with a clammy perspiration. Respiration quick, catching, and at times labored. It was evident that further efforts at medication were useless, and it was decided to watch the progress of the case, and if practicable, attempt the use of chloroform and stimulants, if he could swallow them. Mr. Mason, Drs. Cushing and Stevens kindly volunteered to remain, and the following is from the notes furnished by these gentlemen:

10½, P. M. Expression of countenance anxious; eyes wild and staring. Constant discharges of saliva; occasional vomiting of dark colored matter. Extremities cold; congestion of capillaries. Frequent spasmodic twitchings of the limbs and muscles of respiration. Sudden starting as though alarmed. Frequent interruption of breath and voice by spasmodic closure of the glottis. No pulse perceptible at wrist. Little or no difficulty in deglutition. Delirium. Frequently orders all or part of those present out of the room. Talks at times incoherently; addresses absent persons as though before him. Starts suddenly without apparent object. Suddenly approaching him will cause him to start in alarm. Complains of pain in his head and in his left arm, and especially in his neck, but without locating its seat. Senses all exalted; complains if anything perfumed is brought near him.

11, P. M. More quiet. Pulse at wrist perceptible, but feeble and very rapid. Has taken five teaspoonsful of milk punch, one-third brandy. Apply warm flannel to extremities. Constant spitting.

Jan. 31, midnight. No perceptible change, except dilitation of pupils not before observed.

Stimulants continued.

12½, A. M. Has just had a violent convulsion, lasted but a few moments, and followed by great difficulty in breathing. Each expiration accompanied with a groan. Occasional rattling in throat. Pulseless at wrist, and but slight impulse of heart perceptible. Pupils very much dilated and insensible to light.

12.40. Had another convulsion less severe. Has revived somewhat. Vomits occasionally matter like coffee grounds.

1, A. M. For the last twenty minutes has been sinking rapidly. No more convulsions or spasms. Pulsation of carotids just perceptible. Insensible to all external impressions. Small quantities of blood occasionally issue from corners of mouth.

1.20. Occasional slight spasm of glottis: otherwise no change.

1.35. Pulsation of carotids more distinct. A slight impulse can be detected at the wrist. Occasional spasm of glottis more marked.

1.40. Again vomited—matter like coffee grounds—ejected through mouth and nose.

Sank rapidly, and died at 1.45.

*Dr. Flint, Jr.*, remarked that he had seen in the first number of the Pacific Medical Journal, a journal just commenced at San Francisco, a notice that "a man was cured of hydrophobia, in Italy, lately, by swallowing vinegar in mistake for a medicinal potion. A physician of Padua heard this, and tried the remedy on a patient. He gave him a pint of vinegar in the morning, and another at night, which cured him." Dr. F., Jr., inquired if any members of the association had ever seen, heard of, or read of an analagous case, or a case of recovery from that disease.

*Prof. Hamilton* referred Dr. F. to the legislature of the state of New York, who purchased of John M. Crouse, of Hudson, for $2000, a recipe for the cure of hydrophobia. The recipe consisted of the false tongue of a newly foaled colt, verdigris from copper of George IV, or if it is not possible to procure this, the copper of some of the later kings will do, etc.

This was accompanied by innumerable testimonials, cures, etc.

*Case of a Primipara, aged fifteen years and nine months, delivered at full term, of twins, by the forceps, after having been about fifty hours in labor, Puerperal Convulsions having supervened; both infants living, and together with the mother, doing well.* In the practice of Dr. GOULD. Operation by Prof WHITE. Case attended and reported by BENJ. HEATON LEMON, M. D., Assistant Accoucheur to the "Buffalo Lying-in and Foundling Hospital of the Sisters of Charity."

Maria Holland first menstruated at the age of fourteen; the catamenia last appeared in the latter part of May, 1857. She has had good health during gestation, with the exception of swelling of the lower extremities for the last three weeks, which disappeared entirely just previous to labor.

She was taken in the pains of labor Thursday afternoon, Feb. 4th, and attended by Dr. Austin Flint, Jr., at the request of Dr. Gould, whose patient she was. No progress had been made, however, or was making.

Friday afternoon Dr. Gould saw her. Little progress; parts dry, and the os uteri rigid; exhibited nauseating doses of antimony.

At 7, P. M., I first saw her. Nausea was considerable, retching had occurred several times; the pains were frequent and apparently efficient; the mouth of the womb was dilated to about the size of half a dollar; the parts cool, moist, and soft.

The nurse was directed to give one more dose of antimony and report at 9 o'clock.

Attended again at 12, P. M. She had frequent, strong bearing down pains; the "*os*" was dilated to about the size of a dollar piece; the lips thin and membranous; the parts well lubricated and cool; considerable nausea.

Shortly after twelve the pains became less and less frequent and efficient, and at 1 o'clock, A. M., had ceased altogether; she slept till nearly 2 o'clock; on awaking she had a convulsion, followed in five minutes by another, more severe but shorter: scarcely ended, however, when another began. After an interval of three-quarters of an hour's rest, another convulsion; in five minutes more, another: these were of short duration. Chloroform was administered with the apparent effect of shortening and lessening the severity of the paroxysms. After an interval of 15 minutes, a sixth convulsion, which was followed by three-quarters of an hour's calm and sleep, from which she was awakened by a seventh convulsion, followed in ten minutes by an eighth, when she rested half an hour. Each of the preceding paroxysms lasted, about one and a half minutes by the watch. A fit of three minutes' duration succeeded this rest, not as severe, apparently, though of longer duration than the preceding ones. Two more convulsions recurred at an hour's interval.

From this time the patient was kept partially under the influence of chloroform.

She had twelve convulsions from 2 o'clock, A. M., up to 7, A. M. Between these hours she took two drachms of ergot infused in a pint of water. No perceptible effect was produced by it, however, being probably inert.

Under the partial influence of chloroform the patient remained passive, until the arrival of Prof. White and Dr. Newman, at half past 10, A. M.

Dr. White drew off a small quantity of urine; ruptured the membranes; advised cold to the head, one drop of croton oil to be given immediately, and the continuance of the chloroform as before.

Dr. Newman, on testing the urine, *found no albumen.*

Prof. White to return in two hours.

Shortly before twelve slight pains were manifested at intervals. Dr. White, on his return, pushed up the head in order that the waters might drain away with more facility. After this active labor set in, the pains being efficient and at regular intervals, so that by 2 o'clock, P. M., the vertex presented at the "os-externum," when the contractions again ceased, and the patient lay passive till half-past two, when a convulsion succeeded.

Prof. White being again apprised, returned at half-past 3, P. M., and delivered her under chloroform, with the forceps, of a live female child by the head; shortly after, with his hands, of an asphyxiated female child, the feet presenting. Ergot was given, the secundines soon followed, and the womb contracted well. An assistant was directed to keep his hand on the uterine tumor for two hours, to ensure its continuance.

The convulsions came on not so quickly but that the patient could give notice of it. They began first by severe pain in the head, then shaking of the extremities, and, finally, the whole body becoming violently convulsed, the head thrown back, the jaws champing on a cork covered with a rag, to protect the tongue; foaming at the mouth; the jaws towards the close of the fit becoming *set* on the cork; the eyes wide, staring, and strabismic.

The paroxysms would terminate with sobbing and gasping: a deep stupor would follow, with stertorous respiration for some minutes, when the patient would recover sufficiently to speak. Their average duration was about two minutes each, recurring at intervals of from five, ten, fifteen minutes, and an hour.

If chloroform was inhaled just previous to the access of the paroxysm, it would shorten and alleviate its severity if not prevent it entirely; little benefit seemed to be derived from it after the fit was on, respiration being partially suspended.

It may be worthy of remark, that in this case the convulsions twice supervened upon the cessation of labor pains; that the children were born alive, after the length of time in labor, with the addition of convulsions. Dr. White remarked that this was the third time he had delivered with the forceps during convulsions, the children living. With respect to the character of the paroxysms, he thought that although the works made three varieties, the hysterical, epileptic, and apoplectic, yet neither was identical with "*puerperal convulsions*," these having *distinct features as such*, and only to be described as *puerperal*, possessing as in the above case, features of hysteria, of epilepsy, and of apoplexy, with other characters, which must be seen to be appreciated. That albuminuria and convulsions did not coëxist; no albumen being found either before or after delivery.

*Prof. White* related a singular occurrence in the case of a patient (in the family of an apothecary,) who was affected with erysipelas. Contrary to his custom, (the father being an apothecary,) he did not write his prescription. He ordered the external application of the tincture of iodine, and muriated tincture of iron internally. By a singular mistake the patient applied the muriated tincture of iron externally, which was followed by the most singularly happy effects on the disease. Dr. W. asked if any member of the association knew of a case of erysipelas treated by the external application of the muriated tinct. of iron?

*Dr. Rochester* had used it internally in such cases with good effects, but had never seen it applied to the skin.

It was moved and seconded, that owing to the lateness of the hour and the length of Dr. Newman's report, it be passed over for this evening and made the special business of the next meeting of the association. *Carried.*

On motion, the association adjourned.

AUSTIN FLINT, Jr., M. D.,

Secretary *pro tem.*

